# Ticks From Mink and Their Associated Microorganisms in Spain

**DOI:** 10.1155/tbed/9995586

**Published:** 2025-06-25

**Authors:** Ana M. Palomar, Aránzazu Portillo, Asunción Gómez, Madis Põdra, Paula Santibáñez, Cristina Cervera-Acedo, Sonia Santibáñez, Elena López, Manena Fayos, José A. Oteo

**Affiliations:** ^1^Centre of Rickettsioses and Arthropod-Borne Diseases (CRETAV), Infectious Diseases Department, San Pedro University Hospital-Centre of Biomedical Research from La Rioja (CIBIR), Logroño, La Rioja, Spain; ^2^Division of Environmental Services, Tragsatec, Madrid, Spain; ^3^Centro de Recuperación de Fauna Silvestre de Cantabria, Obregón, Cantabria, Spain

**Keywords:** American mink, European mink, Iberian peninsula, tick-borne bacteria, tick-borne protozoal, tick-borne viruses

## Abstract

Wildlife is an important source of emerging zoonotic agents, including tick-borne ones. Wild carnivores such as mink are commonly parasitized by ticks, which are vectors and reservoirs of zoonotic diseases. Besides the importance of these arthropods as potential sources of diseases in mink, and the role of these mammals as reservoirs of infectious diseases, scarce studies of microorganisms of ticks from mink have been performed. In the present work, ticks collected from mink (European mink [*Mustela lutreola*] and American mink [*Neogale vison*]) from 2007 to 2021 in the North of Spain, and their associated microorganisms (bacteria, protozoan, and viruses), were studied. A total of 916 specimens (154 *Ixodes acuminatus*, 761 *Ixodes hexagonus*, and one *Rhipicephalus sanguineus* sensu lato) were processed in 165 pools (31 *I. acuminatus*, 133 *I. hexagonus*, and one *R. sanguineus* s.l.). The microorganisms detected in *I. acuminatus* pools were *Ehrlichia* sp. (8), *Neoehrlichia mikurensis* (4), *Coxiella* spp. (23), *Rickettsiella* spp. (7), and *Ixovirus* spp. (3). In *I. hexagonus* pools, *Coxiella* spp. (131), *Rickettsiella* spp. (5), *Hepatozoon martis* (5), and *Ixovirus* spp. (6) were amplified. Infection with *Coxiella* spp. was found in the *R. sanguineus* s.l. specimen. Mink are involved in the epidemiology of tick-borne microorganisms, including important pathogens. The role of these tick species as vectors and mink as reservoirs of these microorganisms should be further investigated.

## 1. Introduction

At least 60% of the human emerging infectious disease events (the first detection of case/s of emerging infectious diseases in a human population) are zoonotic and are increasing significantly over time [1]. Around 72% of these events have originated in wildlife, a source of diseases that not only threatens human health (zoonotic spillover) but also domestic and other free-ranging animals (cross-species transmission), especially endangered species [1–3]. Thus, the range of host species susceptible to some of the zoonosis that are current threats to global health, COVID-19 or influenza among others, is increasing and includes several animal species, such as mink, that are not involved in the origin of the pathogens but are involved in their evolution [4–6]. Vector-borne diseases, including those transmitted by ticks, are also responsible for emerging disease events [1]. Wildlife vertebrate species are involved in the enzootic cycles of tick-borne diseases as hosts of tick-vectors (amplifiers and dispersers of ticks) and as hosts, amplifiers and/or reservoirs of tick-borne pathogens. As an example, recent studies have demonstrated that farmed mink also suffer from severe fever with thrombocytopenia syndrome (SFTS), an emerging zoonotic viral tick-borne disease endemic in Asia [7]. Tick-borne diseases represent a worldwide health concern, being ticks the most important vectors of infectious diseases for animals and the second most important for humans, only after mosquitoes [8]. Thus, Crimean-Congo haemorrhagic fever (CCHF), a widespread disease recognized by the World Health Organization as a priority disease for research and development in emergency contexts [9] is worth mentioning.

The European mink (*Mustela lutreola* [Linnaeus 1761]; hereafter EM) is one of the most threatened small carnivores in the world. This mustelid (Carnivora; Mustelidae) is classified as critically endangered worldwide, and only isolated fragmented populations occur in Russia, Estonia, Ukraine, Germany, Romania, France, and Spain [10]. The latest estimation is of just a couple 1000 individuals [11]. The reduction of populations and the range of distribution of the EM in Europe involves several causes, mainly dependent on human intervention, such as the habitat loss/degradation, road-kills, overhunting, and introduction of alien species [12–14]. Regarding the latter matter, the invasive American mink (*Neogale vison* [von Schreber, 1776]; hereafter AM), introduced in Europe for fur farming in the 1920s and by the early 2000s, is one of the main factors in the reduction of EM [14]. The intraguild aggression, but also competition for habitats and food resources, and possibly the introduction of diseases, particularly that caused by the Aleutian Disease Virus, make the AM eradication one of the strengths of the EM conservation programmes [11, 14, 15].

Despite the importance of mustelids as hosts of arthropod vectors, studies of ticks from mink and their associated microorganisms, including those that could adversely affect them, are scarce [7, 16–23]. In the present work, ticks collected in Spain from two mustelid species, the critically endangered EM and the alien AM, and their associated microorganisms were studied.

## 2. Materials and Methods

The current study was carried out from 2007 to 2021 in the north of Spain, in the distribution area of the EM. The Spanish EM population was estimated in 500 specimens in 2014 in territories occupied routinely or sporadically by the AM [11, 13]. In this period, the Spanish Government and Regional Governments implemented different EM conservation programmes, as well as EU-financed programmes were carried out (LIFE GERVE [2007], LIFE Territorio Vison [2010–2015], LIFE LUTREOLA SPAIN/LIFE13NAT/ES/001171 [2014–2019]; in the frame of “Estrategia Nacional de Conservación del Visón Europeo” [MMARM, 2009]). During the different interventions carried out in these projects, live-trapping was used as a principal method for EM monitoring and AM culling. Captured EM were anesthetized and clinically examined in accordance to the Spanish Policy for Animal Protection RD 53/2013, Law 32/2007 and Law 6/2013 (which meets the European Union legislation), and when possible, they were inspected for the presence of ticks. After recovering from anaesthesia, the specimens were released at the capture site. AM were also anesthetized, and after data collection when the specimens were still under anesthesia, they were sacrificed following the welfare legal standards [15].

Ticks were removed from anesthetized EM individuals or from the AM carcasses using forceps. The inspection of captured mink specimens, as well as the removal and storage of ticks, was carried out in five wildlife rescue centres (Martioda [(Alava], Cabárceno [Cantabria], La Fombera [La Rioja], Ilundain [Navarra] and La Alfranca [Zaragoza]) by a responsible veterinarian, assisted by a field technician. Tick specimens from each host were collected separately. Data about the identification of the mink specimens, the area, and the date of collection were recorded. From 2007 to 2014, all the ticks were preserved in ethanol 70%. From 2015 to 2021, they were kept alive until tick identification, and immediately frozen at −80°C (or sporadically preserved in ethanol 70% if they were not received in the lab within seven days after collection).

Ticks were classified using taxonomic keys [24, 25]. Morphological identification was confirmed in selected specimens using molecular biology techniques (Table S1) [26, 27].

Ticks were pooled according to the species, stage, host, area, and preservation method (ethanol/frozen). Tick individuals were surface-sterilized by immersion in benzalkonium chloride 0.1% (5 min), ethanol 70% (1 min), rinsed twice in sterile deionized water and air-dried. Each pool containing immature specimens or halves of adult specimens was used for nucleic acid extraction. Those pools containing specimens preserved in ethanol were only processed for DNA extraction using the DNeasy Blood and Tissue kit (Qiagen, Hilden, Germany) following the manufacturer's recommendations with an overnight incubation. Pools with frozen samples were homogenized in 300 μL of Dulbecco's Modified Eagle Medium (DMEM) with 10,000 units penicillin and 10 mg streptomycin/mL (Sigma, USA). DNA was obtained from one-third of the homogenate as mentioned above. In addition, RNA was extracted from a 100 μL-aliquot using the RNeasy Mini kit (Qiagen, Hilden, Germany), and the remaining homogenate (100 μL) was preserved at −80°C for future analysis. RNA was retrotranscribed using Omniscript (Qiagen, Hilden, Germany).

Quality of nucleic acid extracts and control of PCR inhibitors were checked using a PCR assay that amplified partial mitochondrial 16S rRNA gene of ticks (Table S1) [26]. Positive samples were then analyzed for the presence of microorganisms. All pools were studied for the presence of bacteria (*Anaplasma*, *Ehrlichia*, *Neoehrlichia*, *Borrelia*, *Rickettsia*, *Coxiella*, *Rickettsiella*, *Francisella*, and *Spiroplasma* species) and protozoa (*Babesia*, *Theileria*, *Hepatozoon*, and *Trypanosoma* species) using specific PCR assays. Moreover, pools from frozen ticks were also analyzed for *Orthonairovirus*, *Flaviviridae*, and *Phenuiviridae* viruses (Table S1) [28–52].

Negative controls of extraction, negative controls of PCR (water instead of nucleic acid extracts) and positive controls containing DNA of *Anaplasma phagocytophilum* [53], *Ehrlichia* sp. [54], *Neoehrlichia mikurensis* [53], *Coxiella* sp. [55], *Rickettsia amblyommatis* [56], *Babesia bigemina* [54], or *Spiroplasma* sp. [54] from the Centre of Rickettsioses and Arthropod-Borne Diseases (CRETAV) collection (“Zoonosis collection” registered in the National Registry of Biobanks from Carlos III Health Institute [Reference: C.0006409], located at CRETAV-CIBIR [Biomedical Research Center of La Rioja], Spain) or synthetic DNA from *Coxiella burnetii* or *Francisella tularensis* (AMPLIRUN, Vircell, Spain) or DNA/RNA extracts of *Borrelia spielmannii*, *Trypanosoma brucei*, *Orthoflavivirus japonicum* or *Uukuniemi uukuniemiense*, or lyophilized sera specimens from CCHF-infected humans, kindly provided by colleagues (please see acknowledgements section), were used.

The prevalence of infection was calculated using the minimum infectious rate (MIR), assuming that each positive pool contained only one positive tick, as follows:

  $$\begin{array}{c} \text{MIR}=\;\left(\text{No. of positive pools}/\text{No. of ticks analyzed}\right)\times 100\text{\% }. \end{array}$$

PCR products were visualized by 1% agarose gel electrophoresis. Amplicons of the expected size were subjected to Sanger sequencing at the Molecular Diagnostic Department (CIBIR, Logroño, Spain). Nucleotide sequences were analyzed using the BLAST search (https://blast.ncbi.nlm.nih.gov/Blast.cgi), and new sequences were submitted to GeneBank (https://submit.ncbi.nlm.nih.gov/). The Clustal Omega online software (https://www.ebi.ac.uk/Tools/msa/clustalo/) was used for multiple sequence alignment. Phylogenetic analyses were conducted with MEGA XI (http://www.megasoftware.net) using the maximum likelihood method, including all sites. The nucleotide substitution model was selected according to the Akaike information criterion implemented in MEGA XI. Confidence values for the individual branches of the resulting trees were determined by bootstrap analysis with 500 replicates.

## 3. Results

**Figure 1:**
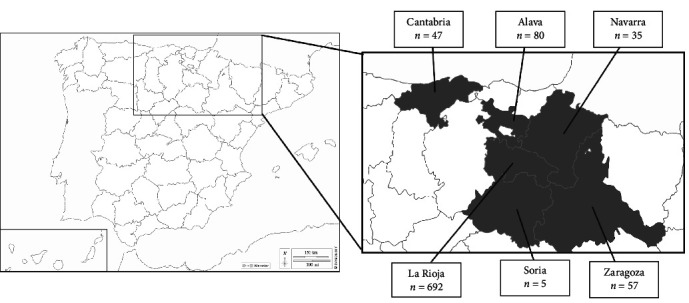
Map of Spain showing the study area (in gray) and the corresponding number of ticks collected from mink in each study area.

A total of 916 ticks were collected from mink in northern Spain from 2007 to 2021: 755 ticks were removed from 147 EM individuals (13 of them were recaptured, 12 twice and one three times), and 161 from 51 AM individuals (Tables S2 and S3). According to the area, 80 were collected in Alava, 47 in Cantabria, 692 in La Rioja, 35 in Navarra, five in Soria and 57 in Zaragoza (Figure 1, Table 1 and Tables S2 and S3). All but one *Rhipicephalus sanguineus* sensu lato specimen belonged to *Ixodes* genus, represented with two species, *Ixodes acuminatus* (*n* = 154) and *Ixodes hexagonus* (*n* = 761). The number of ticks collected in a single EM and AM ranged from 1 to 39 and from 1 to 13, respectively (Tables S2 and S3).

The morphological classification was conclusive for all the samples. Nevertheless, two *I. acuminatus* and three *I. hexagonus* female specimens were genetically characterized using the 16S rRNA and 12S rRNA fragment genes. On the one hand, the two obtained 16S rRNA sequences of *I. acuminatus* were 99.5% identical to each other. One of these sequences (90% query cover) was homologous, and the other one showed 99.75% identity with available sequences of *I. acuminatus* (GenBank accession number [GenBank acc. No.] OL352912 and MH708166, respectively). There were no available public sequences of the 12S rRNA gene of *I. acuminatus*, and the analysis of the two sequences, identical to each other, showed identities <92% with other *Ixodes* species. On the other hand, the 16S rRNA sequences obtained from *I. hexagonus* were identical with each other and identical to those from public *I. hexagonus* sequences (GenBank acc. No. MW114503 and AF549844). Moreover, one of the 12S rRNA sequences belonging to this species was homologous to a public *I. hexagonus* sequence (GenBank acc. No. AF081828), while the remaining two sequences showed a single change, reaching 99.7% identity with other public sequences.

Ticks were grouped in 165 pools, 115 corresponding to the specimens preserved in ethanol (*R. sanguineus* s.l.: 1 pool/1 specimen; *I. acuminatus*: 20 pools/92 specimens; *I. hexagonus*: 94 pools/474 specimens) and 50 corresponding to those preserved frozen (*I. acuminatus*: 11 pools/62 specimens; *I. hexagonus*: 39 pools/287 specimens) (Table 1). Individual male specimens and pools including 5–11 larvae, 2–21 nymphs, 1–12 *I. acuminatus* females, and 1–6 *I. hexagonus* females were processed.

All the pools gave positive results in the 16S rRNA-PCR assays and were analyzed for the microorganism screening.  Table 2 summarizes the obtained results according to host, tick species and stage, and area. The detailed data obtained in each pool are shown in the Table S4.

**Figure 2:**
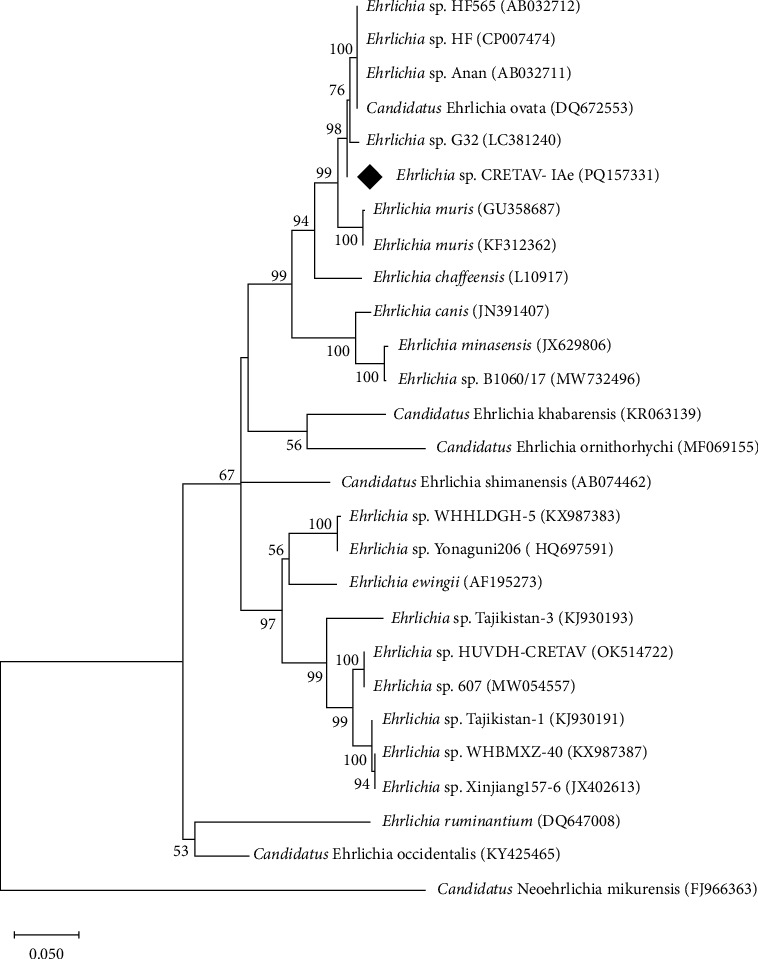
Unrooted dendrogram showing the phylogenetic position of *Ehrlichia* sp. detected in the present study (♦) in *Ixodes acuminatus* female ticks removed from mink among other *Ehrlichia* species. Phylogeny was inferred from comparison of *gro*ELS sequences by the neighbour-joining method and Tamura Nei-model (500 replicates) with a discrete gamma distribution. This analysis involved 27 nucleotide sequences and gaps and missing data were included in the analysis, a total of 1066 positions in the final dataset. *Neoehrlichia mikurensis* was used as outgroup. GenBank accession numbers of the gene used in the comparison are shown in brackets following each species. Replicate numbers of <60% are not shown.

*Ehrlichia* sp. was amplified from 25.8% of the *I. acuminatus* pools (*n* = 8 from La Rioja; Table 2 and Table S4). Identical sequences were obtained for each of the four fragment genes amplified in all these pools, and the analysis of the nucleotide sequences showed the highest identity with *Ehrlichia* sp. HF [57]. Specifically, *gro*ESL, *gltA*, 16S rRNA, and *rpoB* gene fragments reached identities of 99.21%, 97.48%, 99.59%, and 97.57%, respectively, with the available complete genome of *Ehrlichia* sp. HF (GenBank acc. No. CP007474), proposed as *Ehrlichia japonica* sp. nov. [58]. The *gltA* and 16S rRNA genes were homologous (100% identity) with partial sequences of this strain (GenBank acc. No. DQ647319 and DQ647318, respectively). The phylogenetic analysis of *gro*ESL fragment gene supported the clustering with *Ehrlichia* sp. HF (Figure 2).

*N. mikurensis* was detected in four (from La Rioja) out of 31 *I. acuminatus* pools (12.9%) (Table 2 and Table S4). The same genotype was amplified in all the positive pools. The obtained *gro*EL sequences were identical to that detected in a patient from Spain (GenBank acc. No. OQ579033 [59]), an isolate related to Asian strains (GenBank acc. No. MN701626 [60]). The 305 bp 16S rRNA fragment gene of this human isolate from Spain (GenBank acc. No. OQ581737) was also homologous to the one amplified herein. Taking into account a query cover >90%, the analysis of the 16S rRNA fragment gene showed 100% identity with sequences obtained from European ticks different from *I. acuminatus* (GenBank acc. No. KF155493, OP269536, and KF155487, among others), and with 100% query cover they showed a 99.9% identity with sequences from Europe and Asia, including sequences from humans (GenBank acc. No. MN736127, KU535862, CP089286, and CP066557, among others). The *rpoB* sequences showed a 98.8% as highest identity with any of the scarce available sequences from GenBank (only those with complete genome: CP089285-6, CP066557, CP054597, and CP060793).

**Figure 3:**
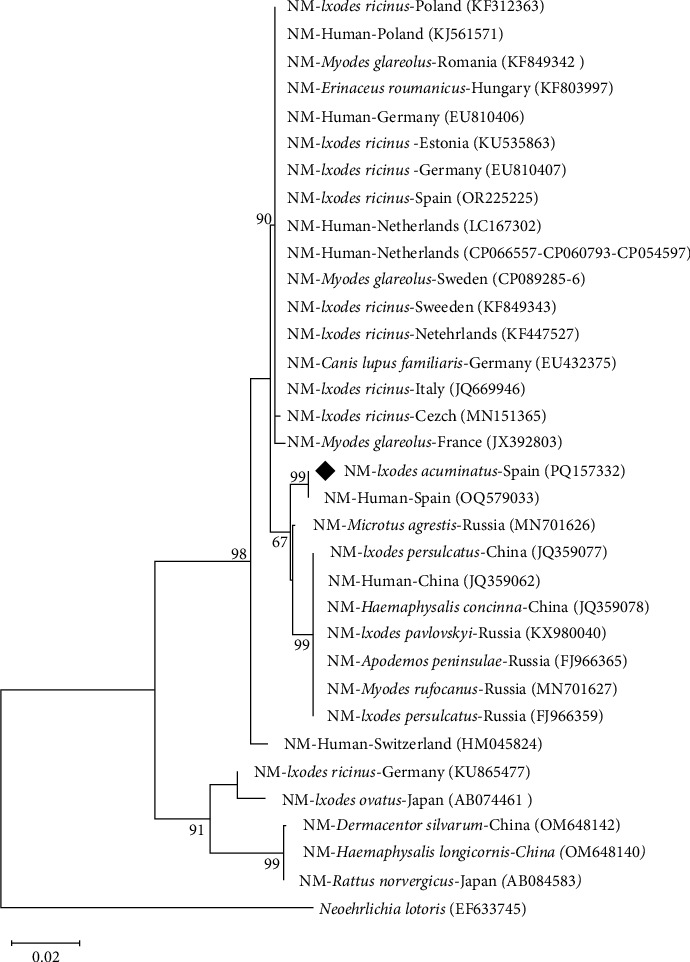
Unrooted dendrogram showing the phylogenetic position of *Neoehrlichia mikurensis* detected in the present study (♦) in *Ixodes acuminatus* female ticks removed from mink among other *N. mikurensis* isolates (NM). Phylogeny was inferred from comparison of *gro*EL sequences by the neighbour-joining method and Tamura Nei-model (500 replicates) with a discrete gamma distribution. This analysis involved 34 nucleotide sequences and gaps and missing data were included in the analysis, a total of 1233 positions in the final dataset. *Neoehrlichia lotoris* was used as outgroup. GenBank accession numbers of the gene used in the comparison are shown in brackets following each species. Replicate numbers of <60% are not shown.

A maximum likelihood tree based on *gro*EL sequences was inferred. The sequences obtained in this study formed an independent cluster with sequences obtained from a patient that suffered for neoehrlichiosis from Spain (GenBank acc. No. OQ579033 [59]) (Figure 3).

*Coxiella* and/or *Rickettsiella* were detected in all but two *Ixodes* pools (30 *I. acuminatus* and 132 *I. hexagonus* pools), in all the studied areas, and in the *R. sanguineus* s.l. sample using the selected PCR assays targeting 16S rRNA, *rpoB*, and *gro*EL genes (Table 2 and Table S4).

*Coxiella* was found in the *R. sanguineus* s.l. pool, in 23 pools of *I. acuminatus* and in 131 pools of *I. hexagonus*. The three PCR target genes (16S rRNA, *rpoB*, and gro*EL*) showed amplicons of the expected size for *R. sanguineus* s.l. Unfortunately, only *rpoB* sequences were readable and showed 99.7% identity with an uncultured *Coxiella* sequence obtained from a questing *R. sanguineus* s.l. found in the same area (Unpublished data, GenBank acc. No. OM302467). According to the sequences of the 16S rRNA (*n* = 29), *rpoB* (*n* = 138), and gro*EL* (*n* = 137) gene fragments, similar *Coxiella* genotypes were detected in both *Ixodes* species. The 29 sequences of the 16S rRNA gene fragment were identical to each other and identical to *Coxiella* endosymbiont sequences found in *Ixodes ricinus* (GenBank acc. No. KP994826 and KP994825) and showed two changes with those available from *Coxiella* endosymbiont of *I. hexagonus* (GenBank acc. No. KP994823 and KP994824). All but one of the *rpoB* sequences (*n* = 137) were identical to each other and indistinguishable (100% identity) from *Coxiella* endosymbionts of *I. hexagonus* (GenBank acc. No. KP985318, KP985319 and MK248731) and *I. ricinus* (GenBank acc. No. KP985320 and KP985321). The remaining *rpoB* sequence showed a single point mutation. All the 137 gro*EL* sequences were homologous and showed 100% identity with *Coxiella* endosymbiont single sequences of *I. ricinus* and *Ixodes uriae* (GenBank acc. No. KP985502 and KJ459059, among others) and showed a single change with those available from *I. hexagonus* (GenBank acc. No. KP985500 and KP985501). Sequences of *Coxiella* amplified from *I. acuminatus* were not available for any of the primer pairs.

*Rickettsiella* sp. was detected in seven *I. acuminatus* and five *I. hexagonus* pools, based on the analysis of five 16S rRNA, one *rpoB* and 11 *gro*EL nucleotide sequences obtained. The 16S rRNA sequences were homologous to that from *Candidatus* Rickettsiella isopodorum (GenBank acc. No. JX406180). The *rpoB* sequence showed the highest identity (90.4%) with *Rickettsiella melolonthae* (GenBank acc. No. EF694042). The analysis of the *gro*EL sequences, all identical with each other, revealed that they were homologous to others previously found in *Ixodes trianguliceps*, *Ixodes ventalloi*, and *Ixodes arboricola* (GenBank acc. No. KY678003, KY677996, and KY677997, among others). Neither *Rickettsiella* sequences from *I. hexagonus* nor from *I. acuminatus* were available.

*Anaplasma*, *Borrelia*, *Rickettsia*, *Francisella*, or *Spiroplasma* species were not amplified in any samples.

*Hepatozoon martis* was the unique protozoan detected. It was found in five *I. hexagonus* pools from La Rioja, one formed with nymphs and four with females (Table 2 and Table S4). The 18S RNA sequences were identical with each other, reaching 100% of identity with the public sequences of *H. martis* (GenBank acc. No. EF222257 and KU198330, among others) amplified from mustelids from different European countries, including Spain, such as *Martes foina* (MG136688), *Martes martes* (GenBank acc. No. MG136687, OM256567, EF222257, and EU686690) and *Meles meles* (GenBank acc. No. KU198330), and from a *Haemaphysalis concinna* tick from Hungary (GenBank acc. No. OM256566).

*Neither Trypanosoma*, *Babesia*, or *Theileria* species were amplified in any samples.

Regarding viruses, the selected PCR assay for the *Orthonairovirus* detection yielded bands of the expected size in 32 out of 50 pools. Unfortunately, only a few sequences were readable, and the tick genome was detected (instead of the viruses). No *Flaviviridae* were detected using the selected PCR assays.

**Figure 4:**
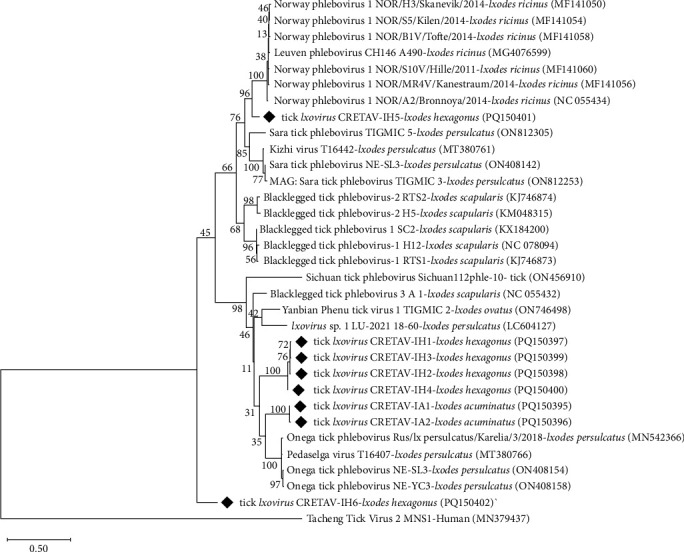
Unrooted dendrogram showing the phylogenetic position of *Ixovirus* spp. detected in the present study (♦) in ticks removed from mink among other isolates. Phylogeny was inferred from comparison of RNA-dependent RNA-polymerase gene (L segment) sequences by the neighbour-joining method and Tamura Nei-model (500 replicates) with a discrete gamma distribution. This analysis involved 33 nucleotide sequences and gaps and missing data were included in the analysis, a total of 503 positions in the final dataset. Tacheng Tick Virus 2 (*Uukuvirus tachengense*) was used as outgroup. GenBank accession numbers of the gene used in the comparison are shown in brackets following each species. Replicate numbers of <60% are not shown.

The two single PCR assays performed for the amplification of the L segment of the *Phenuiviridae* viruses yielded negative results. Using the same primer pairs in a semi-nested assay designed for this study (Table S1), positive results were obtained for 12 pools from La Rioja (Table 2 and Table S4). After sequence analysis, the presence of phenuiviruses was confirmed in nine out of 12 pools, three *I. acuminatus* female, and six *I. hexagonus* female pools. Sequences showed eight different genotypes, two of them from *I. acuminatus* pools (IA1 was amplified in two pools, and IA2 in one pool) and six from *I. hexagonus* pools (IH1-IH6, one per pool). The nucleotide and amino acid sequences reached the highest identities (76.1%−87.5% and 81.5%−98.9%, respectively) with *Ixovirus* spp. sequences previously detected in different *Ixodes* spp. (Tables 3 and 4 and Figure 4), not in *I. acuminatus* or in *I. hexagonus*. The nucleotide sequences of IA1 and IA2 (from *I. acuminatus*) showed a silent point mutation. Four of the six genotypes (IH1−IH4) found in *I. hexagonus* were closely related (≥98% and 99.4% identity for nucleotides and proteins). Genotypes IH5 and IH6 (from *I. hexagonus*) were not similar to each other or to any of the other detected genotypes (Tables 3 and 4). The inferred phylogeny showed two clusters close to *Ixovirus ixodis* (Blacklegged tick phlebovirus 3), one containing the IA genotypes and the other one with four of the IH genotypes (IH1, IH2, IH3, and IH4). Moreover, the genotype IH5 clustered with *Ixovirus norvergiae* (Norway phlebovirus 1), but separately. Lastly, the genotype IH6 belonged to *Ixovirus* spp. but was independent and far from all known species (Figure 4).

Following the International Committee on Taxonomy of Viruses (ICTV) species demarcation criteria (*RdRP* amino acid sequence >95% [61]), only genotype IH5 was identified at the species level, as *I. norvergiae*, and the remaining genotypes belonged to three potential new *Ixovirus* species (1. IA1 and IA2; 2. IH1, IH2, IH3, and IH4; 3. IH6). We propose the following names for these potential new species: *Ixovirus vison* for genotypes IA1-IA2, *Ixovirus beronensis* for genotypes IH1–IH2–IH3–IH4 and *Ixovirus cretavensis* for genotype IH6.

## 4. Discussion

To the best of our knowledge, the present work is the largest study of ticks from mink and their associated microorganisms. A total of 916 ticks collected from EM or AM during 15 years in the North of Spain have been analyzed for the presence of tick-borne bacteria, protozoan and viruses.

Apart from a single specimen of *R. sanguineus* s.l., the most prevalent tick species found infesting EM and AM in the North of Spain have been *I. acuminatus* and *I. hexagonus*. These two species, widely distributed in the Palearctic area, are common parasites of wild carnivores, including mustelids, and have been sporadically described biting humans. The later species also parasitize companion animals such as dogs and cats increasing the risk of exposure to pet owners [25, 62]. *I. acuminatus* and *I. hexagonus* have been previously reported in scarce specimens of AM and EM from Spain [16] and from other European countries [17, 19], and similarly with those studies, immature and adult specimens of the latter tick species have been found herein and only adult *I. acuminatus* specimens were collected. The host preferences for immature and adult stages of *I. hexagonus* are similar, but could be different for the stages of *I. acuminatus* [25]. The main hosts of *I. acuminatus* are insectivores and rodents, mink prey with which they share the habitat and favor the mink exposition to this tick species, but as our results suggest, preferably to adult specimens. At least five tick species had been found in mink in Europe (*I. acuminatus*, *Ixodes canisuga*, *I. hexagonus*, *I. ricinus*, and *Ixodes rugicollis*) and likewise in the present study, *I. hexagonous* had been the most prevalent [19]. *I. ricinus*, the most widespread tick species in Europe and in the study area, has not been detected in this work, but only a few specimens have been reported from mink [17, 19]. The semiaquatic habitat of mink and the use of dens as burrows are not favorable conditions for the development of exophilic tick species (such as *I. ricinus*) but they favor the presence of nest-dwelling tick species (such as *I. acuminatus* or *I. hexagonus*).

Herein, *Ehrlichia* HF has been amplified in *I. acuminatus* from mink. This Anaplasmataceae bacterium was discovered in *Ixodes ovatus* from Japan and has been recently proposed as new species, named *E. japonica* [57, 58, 63]. Its pathogenicity is still under study, but known virulence factors found in the highly pathogenic *Ehrlichia chaffeensis* affecting humans are also found in *Ehrlichia* HF [58]. Experimentally, this microorganism has been also proved to be highly virulent for immunocompetent mice [64]. Before this study, no *Ehrlichia* had been found *in I. acuminatus*. However, apart from *I. ovatus* in Japan, *Ehrlichia* HF had been detected in *I. ricinus*, *Ixodes aphronophorus* and *R. sanguineus* in Europe, but not in Spain [65–68]. Moreover, it had been detected in rodents and in a sick dog from Japan [63, 69]. A unique study about mink potentially infected with an Anaplasmataceae microorganism has been reported from Spain; unfortunately, it included only two mink specimens, and no sequence data for the microorganism identification was obtained [70]. The presence of this *Ehrlichia* in mink and the role of *I. acuminatus* as vector should be further investigated.

*N. mikurensis* is a tick-borne infectious agent responsible for the disease named neoehrlichiosis [71, 72]. The first human case in Spain, our study area, has been recently reported [59], and the obtained isolate from the clinical sample was identical to that detected in the present study. Unfortunately, the tick vector in this Spanish case was unknown, but the sequences differ from those previously detected in *I. ricinus*, including those available from Spain [53]. *N. mikurensis* had not been previously described in *I. acuminatus*, although it had been amplified in several tick species belonging to different genera (*Ixodes*, *Dermacentor*, *Haemaphysalis*, and *Rhipicephalus*), including *I. hexagonus* (negative in the present study). However, the recognized vectors are *I. ricinus* and *Ixodes persulcatus*, and the vector competence of other species should be demonstrated.

The finding of *Ehrlichia* HF and *N. mikurensis* in *I. acuminatus* ticks from mink infested with *I. hexagonus* or *R. sanguineus* s.l. specimens that were negative for these agents (Table S4) suggests the presence of *E. japonica* and *N. mikurensis* in the ticks instead of the blood meal. Nevertheless, further studies about the role of *I. acuminatus* as vector of these microorganisms are recommended.

Several maternally inherited tick endosymbionts are known. Among them, *Coxiella*-like (LE) and *Rickettsiella*-LE are nutritional providing intracellular bacteria required for tick survival and fitness. Ticks and their symbionts coevolve interdependently, but this association is not necessarily stable, and ancestral symbionts can be replaced by new ones [73]. The replacement of *Coxiella*-LE by *Rickettsiella*-LE in some tick species has been previously suggested [74], and our data support this fact since not previous detection of *Rickettsiella* has been reported in these tick species. Similar genotypes of each corresponding bacteria have been detected in *I. acuminatus* and *I. hexagonus* from mink in the studied area, with a higher prevalence of *Coxiella*-LE. *Coxiella* sp. detected in *I. hexagonus* and *I. acuminatus* belong to the same lineage as other *Coxiella* spp. from different *Ixodes* species, suggesting the ancient and stable coevolution of *Coxiella* endosymbionts with these tick species. Buysse et al. [75] showed tropism of this bacterium in the ovaries and Malpighian tubules in *I. hexagonus*, supporting its vertical (transovarial) transmission and the role of this bacterium as nutritional-providing. *C. burnetii* has been previously detected by qPCR assay in *I. acuminatus* from rodents in Italy [76]. According to our results and our own experience with qPCR assays designed for the *C. burnetii* detection prior to the knowledge of *Coxiella*-LE microorganisms, and the absence of sequencing confirmation in that work, a false *C. burnetii* positive result should be taken into account for that study. The presence of *Rickettsiella* has not been previously reported from these two *Ixodes* species but, identical *gro*EL sequences have been detected in other species of the genus, such as *I. arboricola*, *I. ventalloi*, and *I. trianguliceps* [74]. Thus, *Rickettsiella* is prevalent in *Ixodes* genus, but it is not a microorganism exclusive to ticks and their role in tick functions is unclear [74, 77]. It has been suggested as a facultative nutritional symbiont but also as a reproductive symbiont, due to the possible cause of sex ratio distortions in parthenogenetic *Ixodes woodi* [78].

Until the first description of *H. martis* in European pine marten (*M. martes*) in Spain [79], this protozoon had been mainly found in mustelids through Europe, but also in other carnivores, rodents, and in several artiodactyl species [80, 81]. Moreover, its presence in AM specimens from Canada and in other mustelid species from Japan and Korea has been suggested due to the similarities in morphology, pathology and organ distributions, supported in specific cases by molecular biology [80]. The knowledge about the tick species involved in the transmission, via tick ingestion, of *H. martis* is scarce. A questing *I. ricinus* male and a pool of questing *H. concinna* nymphs have been found infected with *H. martis* in Europe, and a closely related genotype has also been detected in questing *Haemaphysalis longicornis* and *Haemaphysalis hystricis* in Japan [80–82]. The presence of *H. martis* in *I. hexagonus* has not been explicitly published, but it is worth mentioning that small sequence fragments obtained from specimens from foxes in Germany were identical to sequences obtained herein [83]. Our data support the implication of *I. hexagonus* in the epidemiology of this tick-borne microorganism, although their role as vectors remains unknown.

Viruses associated with ticks are widely distributed and are responsible for several emerging human diseases. Among them, the family *Phenuiviridae* includes many zoonotic species such as Dabie bandavirus (SFTS), Heartland virus (Heartland virus disease), Tacheng tick virus 2 (TcTV-2 infection), or Bhanja virus (BHAV infection) [84–88]. Thus, an outbreak of SFTS in farmed mink has been recently reported, and the virus transmission from infected mink to humans has been suggested [7, 89]. Moreover, the family *Phenuiviridae* also contains species with a high zoonotic potential prediction, such as *I. norvergiae* detected in this study [90]. Phenuiviruses have been previously detected in several tick species, although not from *I. acuminatus* neither *I. hexagonus*. *Ixovirus* species have been reported from *I. ricinus* and *Ixodes scapularis* in Europe, although not from the study area, possibly due to the lack of investigations [91, 92]. In the present research, two and six *Ixovirus* genotypes have been amplified in *I. acuminatus* and *I. hexagonus*, respectively. According to the ICTV species demarcation criteria, except for one representative of *I. norvegiae*, all the genotypes obtained in this study belong to novel *Ixovirus* species. Deeper studies should be performed, including the virus isolation, to better characterize these genotypes and to investigate their pathogenic potential.

## 5. Conclusions

This study supports the role of mink as important hosts for *I. acuminatus* and *I. hexagonus* and reports the sporadic infestation of mink by *R. sanguineus* s.l. The present research demonstrates the circulation of *E. japonica* and *I. norvergiae* in Spain, and the existence of potential new *Ixovirus* species (proposed as *I. vison*, *I. beronensis* and *I. cretavensis*) with unknown pathogenicity. More efforts should be made to investigate: (1) the role of *I. acuminatus* as a vector of *E. japonica*, *N. mikurensis*, and *Ixovirus* spp., (2) the role of *I. hexagonus* as a vector of *H. martis* and *Ixovirus* spp., (3) the role of mink as reservoirs of these tick-borne microorganisms, (4) the pathogenic potential of these microorganisms for mink and other animals and their zoonotic potential (already confirmed for *N. mikurensis*), (5) their presence in other areas, tick species or hosts, and (6) the potential of *I. acuminatus* and *I. hexagonus* in the enzootic cycle of tick-borne diseases and in the risk of pathogen spillover and disease emergence.

## Figures and Tables

**Table 1:** Ticks collected from mink in Spain (Alava, Cantabria, La Rioja, Navarra, Soria and Zaragoza) from 2007 to 2021.

Host	Tick species	Tick stage	Area	Preserved in ethanol		Preserved frozen	
				No. of ticks	No. of pools	No. of ticks	No. of pools
EM	*I. acuminatus*	Females	La Rioja	64	14	41	5
			Alava	1	1	1	1
			Zaragoza	1	1	12	2
			Navarra	—	—	4	1
		Males	La Rioja	1	1	—	—
	*I. hexagonus*	Females	Alava	15	6	—	—
			La Rioja	139	47	61	13
			Navarra	—	—	5	2
		Males	Alava	1	1	—	—
			La Rioja	—	—	1	1
		Nymphs	Alava	17	2	6	1
			La Rioja	185	19	91	5
			Navarra	—	—	19	2
			Zaragoza	5	1	—	—
		Larvae	Alava	39	4	—	—
			La Rioja	11	2	16	2
			Navarra	—	—	7	1
			Zaragoza	11	1	—	—
	*R. sanguineus* s.l.	Male	La Rioja	1	1	—	—

AM	*I. acuminatus*	Females	La Rioja	25	3	2	1
			Cantabria	—	—	2	1
	*I. hexagonus*	Females	Cantabria	—	—	21	4
			La Rioja	9	3	19	3
			Soria	—	—	4	1
			Zaragoza	7	2	—	—
		Male	La Rioja	—	—	1	1
		Nymphs	Cantabria	—	—	24	2
			La Rioja	14	2	11	1
			Soria	—	—	1	0^a^
			Zaragoza	16	3	—	—
		Larvae	Zaragoza	5	1	—	—

Abbreviations: AM, American mink; EM, European mink; *I. acuminatus*, *Ixodes acuminatus*; *I. hexagonus*, *Ixodes hexagonus*; *R. sanguineus* s.l., *Rhipicephalus sanguineus* sensu lato.

^a^The nymph specimen collected from an AM in Soria was processed with nymphs from La Rioja.

**Table 2:** Microorganisms detected in ticks from mink in Spain (Alava, Cantabria, La Rioja, Navarra, Soria, and Zaragoza) from 2007 to 2021.

Tick species	Host	Stage	No. of ticks/No. of pools		Microorganisms, No. of positive pools/MIR (%)					
			For bacterial and protozoan screening	For viruses screening	*Ehrlichia* sp.	*N. mikurensis*	*Coxiella* spp.	*Rickettsiella* spp.	*H. martis*	Phenuiviruses
*I. acuminatus*	EM	Females	124/25	58/9	6/4.8	3/2.4	19/15.3	5/4.0	0	2/3.5
	AM	Females	29/5	4/2	2/6.9	1/3.5	3/10.4	2/6.9	0	1/25
		Male	1/1	0	0	0	1/100	0	0	0

*Total I. acuminatus*	—	—	154/31	61/11	8/5.2	4/2.6	23/14.8	7/4.5	0	3/4.8

*I. hexagonus*	EM	Larvae	84/10	23/3	0	0	10/11.9	0	0	0
		Nymphs	323/30	116/8	0	0	30/9.3	0	1/0.3	0
		Females	220/68	66/15	0	0	68/30.9	2/0.9	3/1.4	4/6.1
		Males	2/2	1/1	0	0	2/100	0	0	0
	AM	Larvae	5/1	0	0	0	1/20.0	0	0	0
		Nymphs	66/8	36/3	0	0	7/10.6	2/3.0	0	0
		Females	60/13	44/8	0	0	12/20.0	1/1.7	1/1.7	2/4.6
		Male	1/1	1/1	0	0	1/100	0	0	0

Total *I. hexagonus*	—	—	761/133	287/39	0	0	131/17.2	5/0.7	5/0.7	6/2.1

*R. sanguineus* s.l.	EM	Male	1/1	0	0	0	1/100	0	0	0

Total	—	—	916/165	349/50	8/0.9	4/0.4	155/16.9	12/1.3	5/0.6	9/2.6

Abbreviations: AM, American mink (*Neogale vison*); EM, European mink (*Mustela lutreola*); *H., Hepatozoon*; *I., Ixodes*; MIR, minimum infection rate (No. of positive pools/No. of ticks analyzed); N., *Neoehrlichia*; *R., Rhipicephalus*; s.l., sensu lato.

**Table 3:** Clustal analysis of the nucleotide sequences obtained in this study with each other and with other available sequences of *Ixovirus* species.

Virus	IA1	IA2	IH1	IH2	IH3	IH4	IH5	IH6
Tick *Ixovirus* CRETAV-IA1	100.00	**99.79**	76.84	77.05	76.84	77.26	67.80	69.26
Tick *Ixovirus* CRETAV-IA2	**99.79**	100.00	76.63	76.84	76.63	77.05	67.80	69.26
Tick *Ixovirus* CRETAV-IH1	76.84	76.63	100.00	**99.37**	**99.58**	97.70	69.70	69.05
Tick *Ixovirus* CRETAV-IH2	77.05	76.84	99.37	100.00	99.37	**97.91**	70.13	69.47
Tick *Ixovirus* CRETAV-IH3	76.84	76.63	**99.58**	**99.37**	100.00	97.70	69.70	69.05
Tick *Ixovirus* CRETAV-IH4	77.26	77.05	97.70	97.91	97.70	100.00	69.92	69.05
Tick *Ixovirus* CRETAV-IH5	67.80	67.80	69.70	70.13	69.70	69.92	100.00	73.31
Tick *Ixovirus* CRETAV-IH6	69.26	69.26	69.05	69.47	69.05	69.05	73.31	100.00
Onega tick phlebovirus-MN542366 *I. persulcatus*	**80.81**	**80.81**	**79.74**	**79.96**	**79.74**	79.96	70.17	68.66
*Ixovirus ixodis* Blacklegged tick phlebovirus 3-NC_055432 *I. scapularis*	80.63	80.63	79.29	79.71	79.29	**80.75**	72.25	72.63
*Ixovirus norvegiae* Norway phlebovirus 1-MF141050 *I. ricinus*	70.13	70.34	69.92	70.34	69.92	69.28	**87.50**	72.88
*Ixovirus heckscherense* Blacklegged tick phlebovirus 1-KJ746873 *I. scapularis*	68.86	68.86	69.47	69.47	69.47	69.26	81.36	**76.06**
Blacklegged tick phlebovirus 2-KJ746874 *I. scapularis*	69.70	69.70	69.26	69.26	69.26	69.26	81.36	74.36

*Note:* Identity values (%) are shown in the table. Highest identities are highlighted in bold.

**Table 4:** Clustal analysis of the amino acid sequences obtained in this study with each other and with other available sequences of *Ixovirus* species.

Virus	IA1	IA2	IH1	IH2	IH3	IH4	IH5	IH6
Tick *Ixovirus* CRETAV-IA1	100.00	**100.00**	84.81	85.44	84.81	86.08	70.51	72.78
Tick *Ixovirus* CRETAV-IA2	**100.00**	100.00	84.81	85.44	84.81	86.08	70.51	72.78
Tick *Ixovirus* CRETAV-IH1	84.81	84.81	100.00	**99.37**	98.74	98.73	72.44	72.78
Tick *Ixovirus* CRETAV-IH2	85.44	85.44	**99.37**	100.00	**99.37**	**99.37**	73.08	73.42
Tick *Ixovirus* CRETAV-IH3	84.81	84.81	98.74	**99.37**	100.00	98.73	72.44	72.78
Tick *Ixovirus* CRETAV-IH4	86.08	86.08	98.73	**99.37**	98.73	100.00	73.08	73.42
Tick *Ixovirus* CRETAV-IH5	70.51	70.51	72.44	73.08	72.44	73.08	100.00	78.98
Tick *Ixovirus* CRETAV-IH6	72.78	72.78	72.78	73.42	72.78	73.42	78.98	100.00
Onega tick phlebovirus- QPD01619 *I. persulcatus*	87.34	87.34	**86.16**	**86.79**	**86.16**	**87.34**	71.79	72.78
*Ixovirus ixodis* Blacklegged tick phlebovirus 3-ANC97695 *I. scapularis*	**93.04**	**93.04**	84.91	85.53	84.91	86.08	72.44	75.32
*Ixovirus norvegiae* Norway phlebovirus-ASY03242 *I. ricinus*	71.15	71.15	72.61	73.25	72.61	73.08	**98.09**	78.98
*Ixovirus heckscherense* Blacklegged tick phlebovirus 1-AII01806 *I. scapularis*	69.23	69.23	71.97	72.61	71.97	72.44	91.72	80.89
Blacklegged tick phlebovirus 2-AII01807 *I. scapularis*	69.87	69.87	72.61	73.25	72.61	73.08	92.99	**81.53**

*Note:* Identity values (%) are shown in the table. Highest identities are highlighted in bold.

## Data Availability

Sequences obtained in the present study were submitted to GenBank database under the following accession numbers: Tick: *I. acuminatus*: PQ145543 (12S rRNA gene), PQ144653-4 (16S rRNA gene), *I. hexagonus:* PQ145544-5 (12S rRNA gene), PQ144655 (16S rRNA gene); *Ehrlichia* sp.: PQ151704 (16S rRNA gene), PQ157327 (*gltA*), PQ157331 (*rpoB*), PQ157335 (*gro*ESL); *N. mikurensis*: PQ151705 (16S rRNA gene), PQ157332 (*rpoB*), PQ157336 (*gro*ESL); *Coxiella* spp.: PQ151703 (16S rRNA gene), PQ157328-30 (*rpoB*), PQ157334 (*gro*EL); *Rickettsiella* spp.: PQ151706 (16S rRNA gene), PQ157333 (*rpoB*), PQ157337 (*gro*EL); *Hepatozoon martis*: PQ144754 (18S rRNA gene); *Ixovirus* spp.: PQ150395-402 (L segment).

## Supporting Information

Supporting Information Table S1: Primers pairs used in this study. Table S2: Tick collected from European mink in this study. Table S3: Tick collected from American mink in this study. Table S4: Microorganisms detected in each pool of ticks from mink processed in this study.
