# Supplementary material for: Ticks From Mink and Their Associated Microorganisms in Spain
Source: Transbound Emerg Dis. 2025 Jun 25;2025:9995586. doi: 10.1155/tbed/9995586 (PMC12674882; doi:10.1155/tbed/9995586)
Supplement: Supporting Information — Table S1: Primers pairs used in this study. Table S2: Tick collected from European mink in this study. Table S3: Tick collected from American mink in this study. Table S4: Microorganisms detected in each pool of ticks from mink processed in this study. [file 9995586.f1.pdf]

Supplementary table 1: Primers pairs used in this study.

Organisms	Target gene	Primer sequence (5'→ 3')	Fragment size (bp)	Tm (ºC)	References
**Ticks**	16S rRNA	F:CTGCTCAATGATTTTTTAAATTGCTGTGG<br>R:CCGGTCTGAACTCAGATCAAGT	456	48<br>54	[26]
	12S rRNA[1]	F:AAACTAGGATTAGATACCCT<br>R:AATGAGAGCGACGGGCGATGT	338	51<br>53	[27]
**Pan-bacterial**	16S rRNA	F: AGAGTTTGATCCTGGCTCAG<br>R : ACGGCTACCTTGTTACGACTT	1500	60	[28]
***Anaplasma/Ehrlichia/ Neoehrlichia* spp.**	*gro*ESL	F:AITGGGCTGGTAITGAAAT<br>R:CCICCIGGIACIAIACCTTC	1350	48	[29]
		F:ATWGCWAARGAAGCATAGTC<br>R:CTCAACAGCAGCTCTAGTAGC	1297	55	
	gltA[1]	F :ATGTCTACTGCTGCTTGTGA<br>R :ATGACCAGTATATAACTGACGTG	1098	50	[30,31]
		F :ATGTCTACTGCTGCTTGTGA<br>R :GAGCAGACCAACCAGATGTTC	1030	50	
	16S RNA[1]	F :GTGGCAGACGGGTGAGTAAT<br>R :TGCAGCACCTGTGTAAGGTC	1098	60	[32]
***Neoehrlichia* spp.**	*gro*EL	F :GAAGTATAGTTTAGTATTTTTGTC<br>R :TTAACTTCTACTTCGCTTG	1275	49	[33,34]
		F :GAAGTATAGTTTAGTATTTTTGTC<br>R :ACATCACGTTTCATAGAA	510	49	
		F :AAAGGAATTAGTATTAGAATCTTT<br>R :CTTCCATTTTAACTGCTAA	567	49	
		F :AATATAGCAAGATCAGGTAGAC<br>R :TTAACTTCTACTTCGCTTG	461	49	
***Borrelia* spp.**	*flaB*	F: TAATACGTCAGCCATAAATGC<br>R: GCTCTTTGATCAGTTATCATTC	750	56	[35]
	flaB (Lyme group)	F: AARGAATTGGCAGTTCAATC<br>R: GCATTTTCWATTTTAGCAAGTGATG	497	52	[36]
		F:ACATATTCAGATGCAGACAGAGGTTCTA<br>R:GAAGGTGCTGTAGCAGGTGCTGGCTGT	389	55	
**Spotted Fever group *Rickettsia* spp.**	*ompA*	F:ATGGCGAATATTTCTCCAAAA<br>R:GTTCCGTTAATGGCAGCATCT	631	46	[37,38]
		F:ATGGCGAATATTTCTCCAAAA<br>R:AGTGCAGCATTCGCTCCCCCT	532	48	
	ompB	F:AAACAATAATCAAGGTACTGT<br>R:TACTTCCGGTTACAGCAAAGT	811	55	[39]
***Coxiella/ Rickettsiella/ Francisella* spp.**	*rpoB*[2]	F:GGGCGNCAYGGWAAYAAAGGSGT<br>R:CACCRAAHCGTTGACCRCCAAATTG	619	56	[40]
		F:TCGAAGAYATGCCYTATTTAGAAG<br>R:AGCTTTMCCACCSARGGGTTGCTG	542	56	
	groEL	F:TTTGAAAAYATGGGCGCKCAAATGGT<br>R:CGRTCRCCAAARCCAGGTGC	655	56	
		F:GAAGTGGCTTCGCRTACWTCAGACG<br>R:CCAAARCCAGGTGCTTTYAC	619	56	
Francisella tularensis	TUL4	F :ATGGCGAGTGATACTGCTTG<br>R :GCATCATCAGAGCCACCTAA	250	56	[41]
***Spiroplasma* spp.**	*rpoB*	F:GGNTTTATTGAAACACCATAYGCTC<br>R:GCATGTAATTTATCATCAACCATGTGTG	1443	63 -<br>53	[42]
***Babesia/ Theileria/ Hepatozoon* spp.**	18S rRNA	F:GACACAGGGAGGTAGTGACAAG<br>R:CTAAGAATTTCACCTCTGACAGT	400	51	[43]
***Hepatozoon* spp.[1]**	18S rRNA	F: GCGCAAATTACCCAATT<br>R: TAAGGTGCTGAAGGAGTCGTTTAT	660	60	[44,45]

Organisms	Target gene	Primer sequence (5'→ 3')	Fragment size (bp)	Tm (ºC)	References
***Trypanosoma* spp.**	18S rRNA	F: GCTTGTTTCAAGGACTTAGC<br>R:GACTTTTGCTTCCTCTAATG	~1600 bp	52	[46,47]
		F:CGAACAACTGCCCTATCAGC<br>R:GACTACAATGGTCTCTAATC	904	52	
		F:ACCGTTTCGGCTTTTGTTGG<br>R:ACATTGTAGTGCGCGTGTC	904	52	
Orthonairovirus [3]	S Segment	F:TCTCAAAGAAACACGTGCCGC<br>R:GTCCTTCCTCCACTTGW	400	55	[48]
Flaviviridae [3]	NS5	F:TGYRTBTAYAACATGATGG<br>R:GTGTCCCAICCNGCNGTRTC	269-272	50	[49]
	NS5	F:GTGTCCCAGCCGGCGGTGTCATCAGC<br>R:AACATGATGGGRAARAGRGARAA	252	53	[50]
		F:GTGTCCCAGCCGGCGGTGTCATCAGC<br>R:AARGGHAGYMCDGCHATHTGGT	220	54	
Phenuiviridae [3]	L Segment	F:CAGCATGGIGGICTIAGAGAGAT<br>R:TGIAGIATSCCYTGCATCAT	514	55	[51]
	L Segment	F:GGCTACTTCAARAAYAARGANGA<br>R:CTCTCTCAGICCICCRTGYTG	507	50	[52]
	L Segment	F:GGCTACTTCAARAAYAARGANGA<br>R:TGIAGIATSCCYTGCATCAT	1000	50	This study [4]
		F:CAGCATGGIGGICTIAGAGAGAT<br>R:TGIAGIATSCCYTGCATCAT	514	55	

F: Forward; R: Reverse; bp: base pairs; Tm: melting temperature; N= A/C/G/T; Y= C/T; K: G/T; W:A/T; R: G/A;B: C/G/T; S:G/C; H: A/C/T; [1]:Performed only with selected samples; [2.] Also amplified *Ehrlichia* spp. and *Neoehrlichia mikurensis* [3]:Performed only with pools formed by samples that were preserved frozen; [4]: Seminested PCR assay using primers previously designed for single tools [51,52].

Supplementary table 2: Tick collected from European mink in this study.

Mink code	Area	Year of collection	No. of ticks	*I. hexagonus*; No. & Stage	*I. acuminatus*; No. & stage	*R. sanguineus* s.l.; No. & stage
VE1	Zaragoza	2009	1		1F	
VE2	Álava	2008	2	2L		
VE3	Álava	2009	1	1N		
VE4	Álava	2009	1	1N		
VE5	Álava	2009	9	2L;7N		
VE6	Álava	2009	13	6N;5F;1M	1F	
VE7	Álava	2009	1	1F		
VE8	Álava	2009	5	5L		
VE9	Álava	2010	9	1N;8F		
VE10	Álava	2010	1	1F		
VE11	Álava	2010	32	30L;1N;1F		
VE12	La Rioja	2007	1	1F		
VE13	La Rioja	2007	1	1N		
VE14	La Rioja	2007	1	1F		
VE15	La Rioja	2008	18	4N;14F		
VE16	La Rioja	2008	3	3N		
VE17	La Rioja	2008	1	1F		
VE18	La Rioja	2008	3	1N;2F		
VE19	La Rioja	2009	1	1F		
VE20	La Rioja	2009	7	7F		
VE21	La Rioja	2009	10	5N;5F		
VE22	La Rioja	2009	4	1N;1F	2F	
VE23	La Rioja	2009	3	1N;1F	1F	
VE24	La Rioja	2009	4	2F	2F	
VE25	La Rioja	2009	1	1N		
VE26	La Rioja	2009	1	1N		
VE27	La Rioja	2011	8	2L;6N		
VE28	La Rioja	2011	8	3N;3F	2F	
VE29	La Rioja	2011	9	4L;2N;2F	1F	
VE30	La Rioja	2011	2		2F	
VE31	La Rioja	2011	6	4N;2F		
VE32	La Rioja	2011	8	2N;6F		
VE33	La Rioja	2011	3	3F		
VE34[1]	La Rioja	2011	3	2N;1F		
VE36	La Rioja	2008	3	2N;1F		
VE37	La Rioja	2009	3	2N;1F		
VE38	La Rioja	2011	2	1N;1F		
VE39	La Rioja	2011	1	1F		
VE40	La Rioja	2011	3	3L		
VE41	La Rioja	2011	4	4F		
VE42	La Rioja	2011	5	3N;2F		
VE43	La Rioja	2012	2	2N		

Mink code	Area	Year of collection	No. of ticks	*I. hexagonus*; No. & Stage	*I. acuminatus*; No. & stage	*R. sanguineus* s.l.; No. & stage
VE44	La Rioja	2012	3	2N	1F	
VE45	La Rioja	2008	8	1L;4N;3F		
VE46[2]	La Rioja	2008	2	2N		
VE47	La Rioja	2008	2	1F	1F	
VE48	La Rioja	2010	1	1F		
VE49	La Rioja	2010	2	2N		
VE50	La Rioja	2011	1	1F		
VE51	La Rioja	2011	10	5F	5F	
VE52	La Rioja	2007	1	1L		
VE53	La Rioja	2011	2	2F		
VE54	La Rioja	2009	1	1F		
VE55	La Rioja	2007	2	1N	1F	
VE56	La Rioja	2007	1		1F	
VE57	La Rioja	2008	2	1N	1F	
VE58	La Rioja	2008	7	1F	5F;1M	
VE59[3]	La Rioja	2008	2		2F	
VE60	La Rioja	2008	3	1N;2F		
VE61[4]	La Rioja	2008	11	11N		
VE62	La Rioja	2009	2	1N	1F	
VE63	La Rioja	2009	13	10N;3F		
VE64	La Rioja	2009	2	1N;1F		
VE65	La Rioja	2011	11	9N;2F		
VE66	La Rioja	2011	5	5N		
VE67	La Rioja	2011	6	3N;3F		
VE68	La Rioja	2011	4	4F		
VE69	La Rioja	2014	1	1F		
VE70	La Rioja	2014	3	3F		
VE71	La Rioja	2014	10	2L;3N;5F		
VE72	La Rioja	2014	1	1N		
VE73	La Rioja	2014	1	1N		
VE74	La Rioja	2014	6	2N;2F	2F	
VE75	La Rioja	2017	23	7N;1M;14F	1F	
VE76	La Rioja	2017	3	3F		
VE77	La Rioja	2017	2	2F		
VE78	La Rioja	2017	3	1N;1F	1F	
VE79	La Rioja	2013	1		1F	
VE80	La Rioja	2013	7	4F	2F	1M
VE81	La Rioja	2012	1	1N		
VE82	La Rioja	2013	1	1F		
VE83	La Rioja	2012	1		1F	
VE84	La Rioja	2014	3	1N;2F		
VE85	La Rioja	2014	1	1F		
VE86	La Rioja	2012	1	1F		

Mink code	Area	Year of collection	No. of ticks	*I. hexagonus*; No. & Stage	*I. acuminatus*; No. & stage	*R. sanguineus* s.l.; No. & stage
VE87	La Rioja	2013	8	8N		
VE88	La Rioja	2012	8	3N	5F	
VE89	La Rioja	2012	1	1F		
VE90	La Rioja	2014	6	3N;2F	1F	
VE91	La Rioja	2012	6	1N	5F	
VE92	La Rioja	2014	3	2F	1F	
VE93	La Rioja	2013	3	2N;1F		
VE94[5]	La Rioja	2013	4	1N;3F		
VE95	La Rioja	2013	9	3F	6F	
VE96[6]	La Rioja	2014	39	36N;3F		
VE97	La Rioja	2014	10	10N		
VE98[7]	La Rioja	2013	16	5N	11F	
VE99	La Rioja	2014	2	2F		
VE100	La Rioja	2017	3	2N;1F		
VE101	La Rioja	2017	7	6F	1F	
VE102[8]	La Rioja	2013	15	11N;4F		
VE103	La Rioja	2017	3	3L		
VE104	La Rioja	2017	1	1N		
VE105	La Rioja	2017	1	1F		
VE106	La Rioja	2018	1		1F	
VE107	La Rioja	2018	1		1F	
VE108	La Rioja	2019	3	3N		
VE109	La Rioja	2019	6	6N		
VE110	La Rioja	2021	11		11F	
VE111	La Rioja	2019	8	7N;1F		
VE112	La Rioja	2019	4	2N;2F		
VE113	La Rioja	2019	2	2F		
VE114	La Rioja	2020	2	2F		
VE115	La Rioja	2020	1	1F		
VE116	La Rioja	2020	8	7N;1F		
VE117	La Rioja	2020	4	1F	3F	
VE118	La Rioja	2020	5	4L	1F	
VE119	La Rioja	2020	22	1L;20N;1F		
VE120	La Rioja	2020	1	1N		
VE121	La Rioja	2020	5		5F	
VE122	La Rioja	2020	2	1N;1F		
VE123	La Rioja	2020	1	1F		
VE124	La Rioja	2020	4		4F	
VE125	La Rioja	2020	1	1F		
VE126	La Rioja	2020	2		2F	
VE127	La Rioja	2020	2	2F		
VE128	La Rioja	2020	3	2L;1N		
VE129	La Rioja	2020	2	2F		

Mink code	Area	Year of collection	No. of ticks	*I. hexagonus*; No. & Stage	*I. acuminatus*; No. & stage	*R. sanguineus* s.l.; No. & stage
VE130[9]	La Rioja	2020	2	2F		
VE131[10]	La Rioja	2021	1	1F		
VE132	La Rioja	2021	1	1F		
VE133[11]	La Rioja	2021	1	1F		
VE134[12]	La Rioja	2021	1	1N		
VE135	La Rioja	2021	2	2L		
VE136	La Rioja	2021	2	1F	1F	
VE137	La Rioja	2021	4	1F	3F	
VE138	La Rioja	2021	3	2F	1F	
VE139	La Rioja	2021	2	1L;1N		
VE140[13]	La Rioja	2021	21	17N;4F		
VE141[14]	La Rioja	2021	10	1L;7N;1F	1F	
VE142	La Rioja	2021	2	2F		
VE143	La Rioja	2021	5	2N;1F	2F	
VE144	La Rioja	2021	1	1F		
VE145	La Rioja	2021	1	1F		
VE146	La Rioja	2021	1		1F	
VE147	La Rioja	2021	2		2F	
VE148	Álava	2017	5	5N		
VE149	Álava	2015	1	1N		
VE150	Álava	2020	1		1F	
VE151	Navarra	2020	1	1N		
VE152	Navarra	2020	8	7N;1F		
VE153	Navarra	2020	18	7L;7N;3F	1F	
VE154	Navarra	2020	2	2N		
VE155	Navarra	2021	1		1F	
VE156	Navarra	2021	5	2N;1F	2F	
VE157	Zaragoza	2012	15	11L;4N		
VE158	Zaragoza	2012	1	1N		
VE159	Zaragoza	2020	1		1F	
VE160	Zaragoza	2020	5		5F	
VE161	Zaragoza	2021	6		6F	

Recaptured specimens, previous mink codes: [1]: VE16; [2]: VE24; [3]: VE23; [4]: VE25; [5]: VE49; [6]: VE74; [7]: VE51; [8]: VE89; [9]: VE129; [10]: VE109; [11]: VE108; [12]: VE119; [13]: VE119 and VE133; [14]: VE132
I.: *Ixodes*; *R.*: *Rhipicephalus;* s.l.: sensu lato; L: Larvae; N: Nymph; F: Female; M: Male

Supplementary table 3: Tick collected from American mink in this study.

Mink code	Area	Year of collection	No. of ticks	*I. hexagonus*; No. & stage	*I. acuminatus*; No. & stage
VA1	Zaragoza	2009	2	2N	
VA2	Zaragoza	2012	1	1N	
VA3	Zaragoza	2012	2	2N	
VA4	Zaragoza	2012	2	2N	
VA5	Zaragoza	2012	2	2N	
VA6	Zaragoza	2012	5	5L	
VA7	Zaragoza	2012	3	3N	
VA8	Zaragoza	2012	5	3F;2N	
VA9	La Rioja	2009	2	2N	
VA9B	La Rioja	2011	2	2F	
VA10	Soria	2019	2	1N;1F	
VA11	Soria	2020	3	3F	
VA12	Zaragoza	2019	6	2N;4F	
VA13	La Rioja	2014	6	1N;5F	
VA14	La Rioja	2014	1	1F	
VA15	La Rioja	2014	2	1F	1F
VA16	La Rioja	2014	2	2F	
VA17	La Rioja	2014	8	6N;2F	
VA18	La Rioja	2014	6	6N	
VA19	La Rioja	2012	2	2N	
VA20	La Rioja	2012	4		4F
VA21	La Rioja	2013	3	3N	
VA22	La Rioja	2012	2		2F
VA23	La Rioja	2012	2		2F
VA24	La Rioja	2013	2		2F
VA25	La Rioja	2013	3		3F
VA26	La Rioja	2012	1	1F	
VA27	La Rioja	2013	12	3F	9F
VA28	La Rioja	2013	6	1N;2F	3F
VA29	La Rioja	2014	1	1F	
VA30	La Rioja	2018	4	3F	1F
VA31	La Rioja	2019	4	2N;1M;1F	
VA32	La Rioja	2020	1	1F	
VA33	La Rioja	2020	1	1F	
VA34	La Rioja	2021	1	1F	
VA35	La Rioja	2021	3	2N;1F	
VA36	Cantabria	2020	1	1N	
VA37	Cantabria	2018	1		1F
VA38	Cantabria	2020	6	2N;4F	
VA39	Cantabria	2020	3	1N;2F	
VA40	Cantabria	2020	1	1F	
VA41	Cantabria	2020	2	1N	1F

**Mink code**	**Area**	**Year of collection**	**No. of ticks**	***I. hexagonus*; No. & stage**	***I. acuminatus*; No. & stage**
VA42	Cantabria	2019	1	1F	
VA43	Cantabria	2019	1	1N	
VA44	Cantabria	2019	8	4N;4F	
VA45	Cantabria	2018	2	2F	
VA46	Cantabria	2019	4	1N;3F	
VA47	Cantabria	2019	2	1N;1F	
VA48	Cantabria	2020	13	12N;1F	
VA49	Cantabria	2018	1	1F	
VA50	Cantabria	2021	1	1F	

I.: *Ixodes*; L: Larvae; N: Nymph; F: Female; M: Male

Supplementary table 4: Microorganisms detected in each pool of ticks from mink processed in this study

Pool No.	Mink species	Mink code[1]	Area	Tick No.	Tick species	Stage	Microorganisms detected
1	EM	VE148	Alava	6	*I. hexagonus*	N	*Coxiella* sp.
2	EM	**VE156**	Navarra	2	*I. hexagonus*	N	*Coxiella* sp.
3	EM	**VE156**	Navarra	1	*I. hexagonus*	F	*Coxiella* sp.
4	EM	**VE74;VE75;VE78**	La Rioja	4	*I. acuminatus*	F	*Coxiella* sp.
5	AM	**VA15;VA30**	La Rioja	2	*I. acuminatus*	F	*Coxiella* sp./ *Ixovirus* sp.
6	EM	VE110;**VE138**	La Rioja	12	*I. acuminatus*	F	*Ehrlichia* sp./ *N. mikurensis/ Rickettsiella* sp.
7	EM	**VE136;VE137;VE141;VE143;**VE146;VE147	La Rioja	10	*I. acuminatus*	F	*Coxiella* sp. / *Ixovirus* sp.
8	EM	VE161	Zaragoza	6	*I. acuminatus*	F	*Coxiella* sp.
9	EM	VE155;**VE156; VE153**	Navarra	4	*I. acuminatus*	F	*Coxiella* sp.
10	AM	**VA13;VA17;VA31;VA10; VA35**	La Rioja (11)/ Soria (1)	12	*I. hexagonus*	N	*Rickettsiella* sp. / *Coxiella* sp.
11	AM	**VA31**	La Rioja	1	*I. hexagonus*	M	*Coxiella* sp.
12	AM	**VA13**; VA14;VA29	La Rioja	7	*I. hexagonus*	F	*Rickettsiella* sp ./ *Coxiella* sp. / *Ixovirus* sp.
13	AM	VA15;VA16;**VA17**	La Rioja	5	*I. hexagonus*	F	*Coxiella* sp.
14	AM	**VA30;VA31;**VA32;VA34;**VA35**	La Rioja	7	*I. hexagonus*	F	*Coxiella* sp. / *Ixovirus* sp.
15	EM	**VE71;**VE103;**VE139;VE141;VE135**	La Rioja	9	*I. hexagonus*	L	*Coxiella* sp.
16	EM	**VE71**;VE72;VE73**;VE74;VE75;VE78**;VE104	La Rioja	16	*I. hexagonus*	N	*Coxiella* sp.
17	EM	VE108;VE109**;VE111;VE112**	La Rioja	18	*I. hexagonus*	N	*Coxiella* sp.
18	EM	**VE128; VE116;VE139;VE141;VE143**	La Rioja	18	*I. hexagonus*	N	*Coxiella* sp.
19	EM	VE134;**VE140**	La Rioja	18	*I. hexagonus*	N	*Coxiella* sp.
20	EM	**VE75**	La Rioja	1	*I. hexagonus*	M	*Coxiella* sp.
21	EM	VE69;**VE70;VE74**	La Rioja	4	*I. hexagonus*	F	*Coxiella* sp. / *I. norvegiae*
22	EM	**VE70**	La Rioja	2	*I. hexagonus*	F	*Coxiella* sp.
23	EM	**VE71**	La Rioja	5	*I. hexagonus*	F	*Coxiella* sp. / *Ixovirus* sp.
24	EM	**VE75**	La Rioja	5	*I. hexagonus*	F	*Coxiella* sp.
25	EM	**VE75**	La Rioja	6	*I. hexagonus*	F	*Coxiella* sp.
26	EM	**VE75;VE78;**VE105	La Rioja	5	*I. hexagonus*	F	*Coxiella* sp.

Pool No.	Mink species	Mink code[1]	Area	Tick No.	Tick species	Stage	Microorganisms detected
27	EM	**VE111;VE112**	La Rioja	3	*I. hexagonus*	F	*Coxiella* sp.
28	EM	VE151;**VE152;VE153**;VE154	Navarra	17	*I. hexagonus*	N	*Coxiella* sp.
29	EM	**VE152;VE153**	Navarra	4	*I. hexagonus*	F	*Coxiella* sp.
30	EM	**VE153**	Navarra	7	*I. hexagonus*	L	*Coxiella* sp.
31	EM	VE150	Alava	1	*I. acuminatus*	F	*Coxiella* sp.
32	EM	VE159;VE160	Zaragoza	6	*I. acuminatus*	F	*Coxiella* sp.
33	AM	VA10;**VA11**	Soria	4	*I. hexagonus*	F	*Coxiella* sp.
34	EM	VE118;**VE119;VE128**	La Rioja	7	*I. hexagonus*	L	*Coxiella* sp.
35	EM	**VE119;VE120**	La Rioja	21	*I. hexagonus*	N	*Coxiella* sp.
36	EM	**VE117;VE118;**VE121	La Rioja	9	*I. acuminatus*	F	*Ehrlichia* sp. / *Rickettsiella* sp.
37	EM	VE124;VE126	La Rioja	6	*I. acuminatus*	F	*Coxiella* sp. / *Ixovirus* sp.
38	EM	**VE117;VE119;VE122;**VE123;VE125	La Rioja	5	*I. hexagonus*	F	*Coxiella* sp.
39	EM	**VE116;**VE127;VE129	La Rioja	5	*I. hexagonus*	F	*Coxiella* sp. / *H. martis / Ixovirus* sp.
40	EM	VE114;VE130	La Rioja	4	*I. hexagonus*	F	*Coxiella* sp. /*H. martis / Ixovirus* sp.
41	EM	VE131;VE132;VE133;**VE137**;VE145	La Rioja	5	*I. hexagonus*	F	*Coxiella* sp.
42	EM	**VE136;VE138;** VE142;**VE143**	La Rioja	6	*I. hexagonus*	F	*Coxiella* sp.
43	EM	**VE140;VE141**;VE144	La Rioja	6	*I. hexagonus*	F	*Coxiella* sp.
44	AM	VA37;**VA41**	Cantabria	2	*I. acuminatus*	F	*Rickettsiella* sp. / *Coxiella* sp.
45	AM	VA36;**VA38;VA39;VA41**;VA43;**VA44;VA46; VA47**	Cantabria	12	*I. hexagonus*	N	*Rickettsiella* sp.
46	AM	VA48	Cantabria	12	*I. hexagonus*	N	*Coxiella* sp.
47	AM	VA45; **VA46**;VA49	Cantabria	6	*I. hexagonus*	F	*Coxiella* sp.
48	AM	VA42;**VA44;VA47**	Cantabria	6	*I. hexagonus*	F	*Coxiella* sp.
49	AM	**VA38**	Cantabria	4	*I. hexagonus*	F	*Coxiella* sp.
50	AM	**VA39;**VA40; **VA48;**VA50	Cantabria	5	*I. hexagonus*	F	*Coxiella* sp.
51	EM	VE79**;VE90;VE92;VE101;**VE106;VE107	La Rioja	6	*I. acuminatus*	F	*Rickettsiella* sp.
52	AM	VA20;VA23;VA24;VA25	La Rioja	11	*I. acuminatus*	F	*Ehrlichia* sp. / *Rickettsiella* sp.
53	AM	VA22;**VA27**	La Rioja	11	*I. acuminatus*	F	*Ehrlichia* sp.
54	EM	**VE80;VE95**	La Rioja	8	*I. acuminatus*	F	*Ehrlichia* sp. / *N. mikurensis / Coxiella* sp.
55	EM	**VE98**	La Rioja	11	*I. acuminatus*	F	*Ehrlichia* sp. / *N. mikurensis / Coxiella* sp.
56	EM	**VE83;VE88;VE91**	La Rioja	11	*I. acuminatus*	F	*Ehrlichia* sp. / *Rickettsiella* sp.

Pool No.	Mink species	Mink code[1]	Area	Tick No.	Tick species	Stage	Microorganisms detected
57	AM	**VA28**	La Rioja	3	*I. acuminatus*	F	*N. mikurensis / Coxiella* sp.
58	EM	**VE80**	La Rioja	1	*R. sanguineus* s.l.	M	*Coxiella* sp.
59	EM	VE93	La Rioja	1	*I. hexagonus*	F	*Coxiella* sp.
60	EM	VE113	La Rioja	2	*I. hexagonus*	F	*Coxiella* sp.
61	EM	VE76;VE77;**VE100**	La Rioja	6	*I. hexagonus*	F	*Coxiella* sp.
62	EM	VE81;**VE88;VE91;VE93;VE100**	La Rioja	9	*I. hexagonus*	N	*Coxiella* sp.
63	EM	**VE101**	La Rioja	6	*I. hexagonus*	F	*Coxiella* sp.
64	EM	**VE84**	La Rioja	2	*I. hexagonus*	F	*Coxiella* sp.
65	EM	VE85;**VE90;VE92**	La Rioja	5	*I. hexagonus*	F	*Coxiella* sp. / *H. martis*
66	EM	**VE96;VE99**	La Rioja	5	*I. hexagonus*	F	*Coxiella* sp.
67	EM	**VE84;VE90;**VE97	La Rioja	14	*I. hexagonus*	N	*Coxiella* sp.
68	EM	**VE96**	La Rioja	18	*I. hexagonus*	N	*Coxiella* sp.
69	EM	**VE96**	La Rioja	18	*I. hexagonus*	N	*Coxiella* sp.
70	AM	VA18;VA19;VA21	La Rioja	11	*I. hexagonus*	N	*Coxiella* sp.
71	EM	VE87;**VE94;VE98**	La Rioja	14	*I. hexagonus*	N	*Coxiella* sp.
72	EM	VE102	La Rioja	11	*I. hexagonus*	N	*Coxiella* sp.
73	AM	VA26;VA27;VA33	La Rioja	5	*I. hexagonus*	F	*Coxiella* sp. / *H. martis*
74	EM	**VE80**	La Rioja	4	*I. hexagonus*	F	*Coxiella* sp.
75	EM	VE82;**VE102**	La Rioja	5	*I. hexagonus*	F	*Coxiella* sp.
76	EM	**VE94; VE95**	La Rioja	5	*I. hexagonus*	F	*Coxiella* sp.
77	EM	VE86;VE89**;VE94**	La Rioja	5	*I. hexagonus*	F	*Coxiella* sp.
78	EM	**VE157;**VE158	Zaragoza	5	*I. hexagonus*	N	*Coxiella* sp.
79	EM	**VE157**	Zaragoza	11	*I. hexagonus*	L	*Coxiella* sp.
80	AM	**VA12**	Zaragoza	4	*I. hexagonus*	F	*Coxiella* sp.
81	AM	**VA28**	La Rioja	2	*I. hexagonus*	F	*Coxiella* sp.
82	AM	**VA12**	Zaragoza	2	*I. hexagonus*	N	*Coxiella* sp.
83	AM	VA1;VA2;VA3;VA4	Zaragoza	7	*I. hexagonus*	N	*Coxiella* sp.
84	AM	VA6	Zaragoza	5	*I. hexagonus*	L	*Coxiella* sp.
85	AM	VA5;VA7;**VA8**	Zaragoza	7	*I. hexagonus*	N	*Coxiella* sp.

Pool No.	Mink species	Mink code[1]	Area	Tick No.	Tick species	Stage	Microorganisms detected
86	AM	**VA8**	Zaragoza	3	*I. hexagonus*	F	*Coxiella* sp.
87	EM	VE1	Zaragoza	1	*I. acuminatus*	F	*Coxiella* sp.
88	EM	VE2;**VE5;**VE8	Alava	9	*I. hexagonus*	L	*Coxiella* sp.
89	EM	VE3;VE4;**VE5**	Alava	9	*I. hexagonus*	N	*Coxiella* sp.
90	EM	**VE6**	Alava	1	*I. hexagonus*	M	*Coxiella* sp.
91	EM	**VE6**	Alava	1	*I. hexagonus*	F	*Coxiella* sp.
92	EM	**VE6;VE9; VE11**	Alava	8	*I. hexagonus*	N	*Coxiella* sp.
93	EM	**VE6**	Alava	2	*I. hexagonus*	F	*Coxiella* sp.
94	EM	**VE22**	La Rioja	2	*I. acuminatus*	F	*Coxiella* sp.
95	EM	**VE6;** VE7; VE10;**VE11**	Alava	4	*I. hexagonus*	F	*Rickettsiella* sp. / *Coxiella* sp.
96	EM	**VE9**	Alava	3	*I. hexagonus*	F	*Coxiella* sp.
97	EM	**VE9**	Alava	3	*I. hexagonus*	F	*Coxiella* sp.
98	EM	**VE11**	Alava	10	*I. hexagonus*	L	*Coxiella* sp.
99	EM	**VE11**	Alava	10	*I. hexagonus*	L	*Coxiella* sp.
100	EM	**VE11**	Alava	10	*I. hexagonus*	L	*Coxiella* sp.
101	EM	**VE27;VE29**	La Rioja	6	*I. hexagonus*	L	*Coxiella* sp.
102	EM	VE13; **VE15**; VE16	La Rioja	8	*I. hexagonus*	N	*Coxiella* sp.
103	EM	**VE18; VE21; VE22**	La Rioja	7	*I. hexagonus*	N	*Coxiella* sp.
104	EM	**VE23;**VE25;VE26**;VE27**	La Rioja	7	*I. hexagonus*	N	*Coxiella* sp.
105	EM	**VE27;VE28;VE29**	La Rioja	7	*I. hexagonus*	N	*Coxiella* sp.
106	EM	**VE31:VE32;VE34**	La Rioja	8	*I. hexagonus*	N	*Coxiella* sp.
107	EM	**VE9**	Alava	2	*I. hexagonus*	F	*Coxiella* sp.
108	EM	VE12; VE14; **VE15;VE22**	La Rioja	4	*I. hexagonus*	F	*Coxiella* sp.
109	EM	**VE15**	La Rioja	2	*I. hexagonus*	F	*Coxiella* sp.
110	EM	**VE15**	La Rioja	3	*I. hexagonus*	F	*Coxiella* sp.
111	EM	**VE15**	La Rioja	4	*I. hexagonus*	F	*Coxiella* sp.
112	EM	**VE15**	La Rioja	1	*I. hexagonus*	F	*Rickettsiella* sp. / *Coxiella* sp.
113	EM	**VE15**	La Rioja	3	*I. hexagonus*	F	*Coxiella* sp.
114	EM	VE17; VE19	La Rioja	2	*I. hexagonus*	F	*Coxiella* sp.
115	EM	**VE18**	La Rioja	2	*I. hexagonus*	F	*Coxiella* sp.

Pool No.	Mink species	Mink code[1]	Area	Tick No.	Tick species	Stage	Microorganisms detected
116	EM	**VE20**	La Rioja	1	*I. hexagonus*	F	*Coxiella* sp.
117	EM	**VE20**	La Rioja	2	*I. hexagonus*	F	*Coxiella* sp.
118	EM	**VE20**	La Rioja	2	*I. hexagonus*	F	*Coxiella* sp.
119	EM	**VE20**	La Rioja	2	*I. hexagonus*	F	*Coxiella* sp.
120	EM	**VE21**	La Rioja	2	*I. hexagonus*	F	*Coxiella* sp.
121	EM	**VE21**	La Rioja	2	*I. hexagonus*	F	*Coxiella* sp.
122	EM	**VE23; VE24**	La Rioja	3	*I. hexagonus*	F	*Coxiella* sp.
123	EM	**VE28**	La Rioja	2	*I. hexagonus*	F	*Coxiella* sp.
124	EM	**VE28;VE29**	La Rioja	2	*I. hexagonus*	F	*Coxiella* sp.
125	EM	**VE29;VE31**	La Rioja	3	*I. hexagonus*	F	*Coxiella* sp.
126	EM	**VE32**	La Rioja	2	*I. hexagonus*	F	*Coxiella* sp.
127	EM	**VE32**	La Rioja	3	*I. hexagonus*	F	*Coxiella* sp.
128	EM	VE33;**VE34**	La Rioja	4	*I. hexagonus*	F	*Coxiella* sp.
129	EM	**VE23; VE24;VE28**	La Rioja	4	*I. acuminatus*	F	*Coxiella* sp.
130	EM	**VE28**	La Rioja	1	*I. acuminatus*	F	
131	EM	**VE29;**VE30	La Rioja	3	*I. acuminatus*	F	*Coxiella* sp.
132	EM	**VE44**	La Rioja	1	*I. acuminatus*	F	*Coxiella* sp.
133	AM	VA9, **VA28;**	La Rioja	3	*I. hexagonus*	N	*Coxiella* sp.
134	AM	VA9B	La Rioja	2	*I. hexagonus*	F	
135	EM	VE40;VE52; **VE45**	La Rioja	5	*I. hexagonus*	L	*Coxiella* sp.
136	EM	**VE36;VE37;VE38;**VE43	La Rioja	8	*I. hexagonus*	N	*Coxiella* sp.
137	EM	**VE42;VE44**	La Rioja	5	*I. hexagonus*	N	*Coxiella* sp.
138	EM	**VE37;VE38**;VE39	La Rioja	3	*I. hexagonus*	F	*Coxiella* sp.
139	EM	**VE42;**	La Rioja	2	*I. hexagonus*	F	*Coxiella* sp.
140	EM	**VE47;VE55;**VE56**;VE57**	La Rioja	4	*I. acuminatus*	F	*Coxiella* sp.
141	EM	VE41	La Rioja	4	*I. hexagonus*	F	*Coxiella* sp.
142	EM	**VE51**	La Rioja	4	*I. acuminatus*	F	*Coxiella* sp.
143	EM	**VE51;VE58**	La Rioja	3	*I. acuminatus*	F	*Coxiella* sp.
144	EM	**VE58;**VE59;**VE62**	La Rioja	4	*I. acuminatus*	F	*Coxiella* sp.
145	EM	**VE58**	La Rioja	2	*I. acuminatus*	F	*Coxiella* sp.

Pool No.	Mink species	Mink code[1]	Area	Tick No.	Tick species	Stage	Microorganisms detected
146	EM	**VE58**	La Rioja	1	*I. acuminatus*	M	*Coxiella* sp.
147	EM	**VE6**	Alava	1	*I. acuminatus*	F	*Rickettsiella* sp.
148	EM	**VE45;**VE46:VE49	La Rioja	8	*I. hexagonus*	N	*Coxiella* sp.
149	EM	**VE55;VE57;VE60;VE61;VE62**	La Rioja	7	*I. hexagonus*	N	*Coxiella* sp. / *H. martis*
150	EM	**VE61**	La Rioja	8	*I. hexagonus*	N	*Coxiella* sp.
151	EM	**VE63**	La Rioja	10	*I. hexagonus*	N	*Coxiella* sp.
152	EM	**VE64;**VE66**;VE67**	La Rioja	9	*I. hexagonus*	N	*Coxiella* sp.
153	EM	**VE65**	La Rioja	9	*I. hexagonus*	N	*Coxiella* sp.
154	EM	**VE45;VE47**	La Rioja	4	*I. hexagonus*	F	*Coxiella* sp.
155	EM	VE48;VE50**;VE51**	La Rioja	3	*I. hexagonus*	F	*Coxiella* sp.
156	EM	**VE51**	La Rioja	4	*I. hexagonus*	F	*Coxiella* sp.
157	EM	VE53;VE54;**VE58**	La Rioja	4	*I. hexagonus*	F	*Coxiella* sp.
158	EM	**VE60;VE65**	La Rioja	4	*I. hexagonus*	F	*Coxiella* sp.
159	EM	**VE63;VE64**	La Rioja	4	*I. hexagonus*	F	*Coxiella* sp.
160	EM	**VE67**	La Rioja	3	*I. hexagonus*	F	*Coxiella* sp.
161	EM	**VE68**	La Rioja	2	*I. hexagonus*	F	*Coxiella* sp.
162	EM	**VE68**	La Rioja	2	*I. hexagonus*	F	*Coxiella* sp.
163	EM	**VE21**	La Rioja	1	*I. hexagonus*	F	*Coxiella* sp.
164	EM	**VE32**	La Rioja	1	*I. hexagonus*	F	*Coxiella* sp.
165	EM	**VE36**	La Rioja	1	*I. hexagonus*	F	*Coxiella* sp.

[1]: In bold are highlighted those mink whose ticks were included in more than one pool; EM: European mink (*Mustela lutreola*); AM: American mink (*Neogale vison*); *I. acuminatus: Ixodes acuminatus; I. hexagonus: Ixodes hexagonus; R. sanguineus* s.l.: *Rhipicephalus sanguineus* sensu lato; F: female; M: male; N: Nymph; L: Larvae; *N. mikurensis*: *Neoehrlichia mikurensis*; *H. martis: Hepatozoon martis*; *I. norvegiae: Ixovirus norvegiae*
